# Origin Sample Prediction and Spatial Modeling of Antimicrobial Resistance in Metagenomic Sequencing Data

**DOI:** 10.3389/fgene.2021.642991

**Published:** 2021-03-04

**Authors:** Maya Zhelyazkova, Roumyana Yordanova, Iliyan Mihaylov, Stefan Kirov, Stefan Tsonev, David Danko, Christopher Mason, Dimitar Vassilev

**Affiliations:** ^1^Faculty of Mathematics and Informatics, Sofia University St. Kliment Ohridski, Sofia, Bulgaria; ^2^Department of Mathematics, Hokkaido University, Sapporo, Japan; ^3^Bulgarian Academy of Sciences, Institute of Mathematics and Informatics, Sofia, Bulgaria; ^4^Bristol-Myers Squibb, Pennington, NJ, United States; ^5^Department of Molecular Genetics, AgroBioInstitute, Sofia, Bulgaria; ^6^Department of Computational Informatics, Weill Cornell Medical College, New York, NY, United States; ^7^Weill Cornell Medicine, New York, NY, United States

**Keywords:** metagenomics, antimicrobial resistance, classification, spatial correlation, Bayesian hierarchical models, machine learning

## Abstract

The steady elaboration of the Metagenomic and Metadesign of Subways and Urban Biomes (MetaSUB) international consortium project raises important new questions about the origin, variation, and antimicrobial resistance of the collected samples. CAMDA (Critical Assessment of Massive Data Analysis, http://camda.info/) forum organizes annual challenges where different bioinformatics and statistical approaches are tested on samples collected around the world for bacterial classification and prediction of geographical origin. This work proposes a method which not only predicts the locations of unknown samples, but also estimates the relative risk of antimicrobial resistance through spatial modeling. We introduce a new component in the standard analysis as we apply a Bayesian spatial convolution model which accounts for spatial structure of the data as defined by the longitude and latitude of the samples and assess the relative risk of antimicrobial resistance taxa across regions which is relevant to public health. We can then use the estimated relative risk as a new measure for antimicrobial resistance. We also compare the performance of several machine learning methods, such as Gradient Boosting Machine, Random Forest, and Neural Network to predict the geographical origin of the mystery samples. All three methods show consistent results with some superiority of Random Forest classifier. In our future work we can consider a broader class of spatial models and incorporate covariates related to the environment and climate profiles of the samples to achieve more reliable estimation of the relative risk related to antimicrobial resistance.

## 1. Introduction

Antimicrobial resistance (AMR) occurs when bacteria, fungus, and other microorganisms become resistant to antibiotics, antifungals, or other antimicrobial drugs. This leads to persistent infections which are difficult to treat. Such resistance can be achieved both through mutations or through horizontal gene transfer among bacteria from the same or different species (Thomas and Nielsen, 2005). The exposure to antibiotics and other antimicrobial drugs aggravates the problem and leads to many drug-resistant pathogens. Antibiotic resistance genes (ARGs) create a serious health problem that appears not only in clinical settings but also in non clinical environments harboring many resistant bacteria. Resistant bacteria is documented in the human food chain and it may pose significant health risks (Bennani et al., 2020). Many environmental factors such as animal husbandry, waste management, drinking water, and sanitation also contribute to antimicrobial resistance (Fletcher, 2015; Wall, 2019). With the advance of next generation sequencing technologies complex metagenomes are studied. A number of bioinformatics methods and tools exist to analyze such data and discover AMR mechanisms (Lal Gupta et al., 2020; Van Camp et al., 2020). Such mechanisms are subject of intensive research studies which include negative binomial, quasi-Poisson, Zero-inflated models (Hüls et al., 2017). The International Metagenomics and Metadesign of Subways and Urban Biomes (MetaSUB) Consortium is a multidisciplinary initiative with participation of a large number of researchers in different fields who develop and apply metagenomic methods for sample collection, DNA/RNA isolation, taxa characterization, and data visualization (Mason et al., 2016). One of the MetaSUB's goals is to create a global genetic cartography of urban species based on extensive sampling of mass-transit systems and other public areas across the globe. In strategic partnership an extended set of data from global City Sampling Days is first introduced through the annual CAMDA contests. The data of the current challenge consists of a set of over thousand novel samples from 23 cities. Properties related to the climate conditions are also available with the goal of better understanding the relationship between metagenomic profiles and environment.

**Figure 1:**
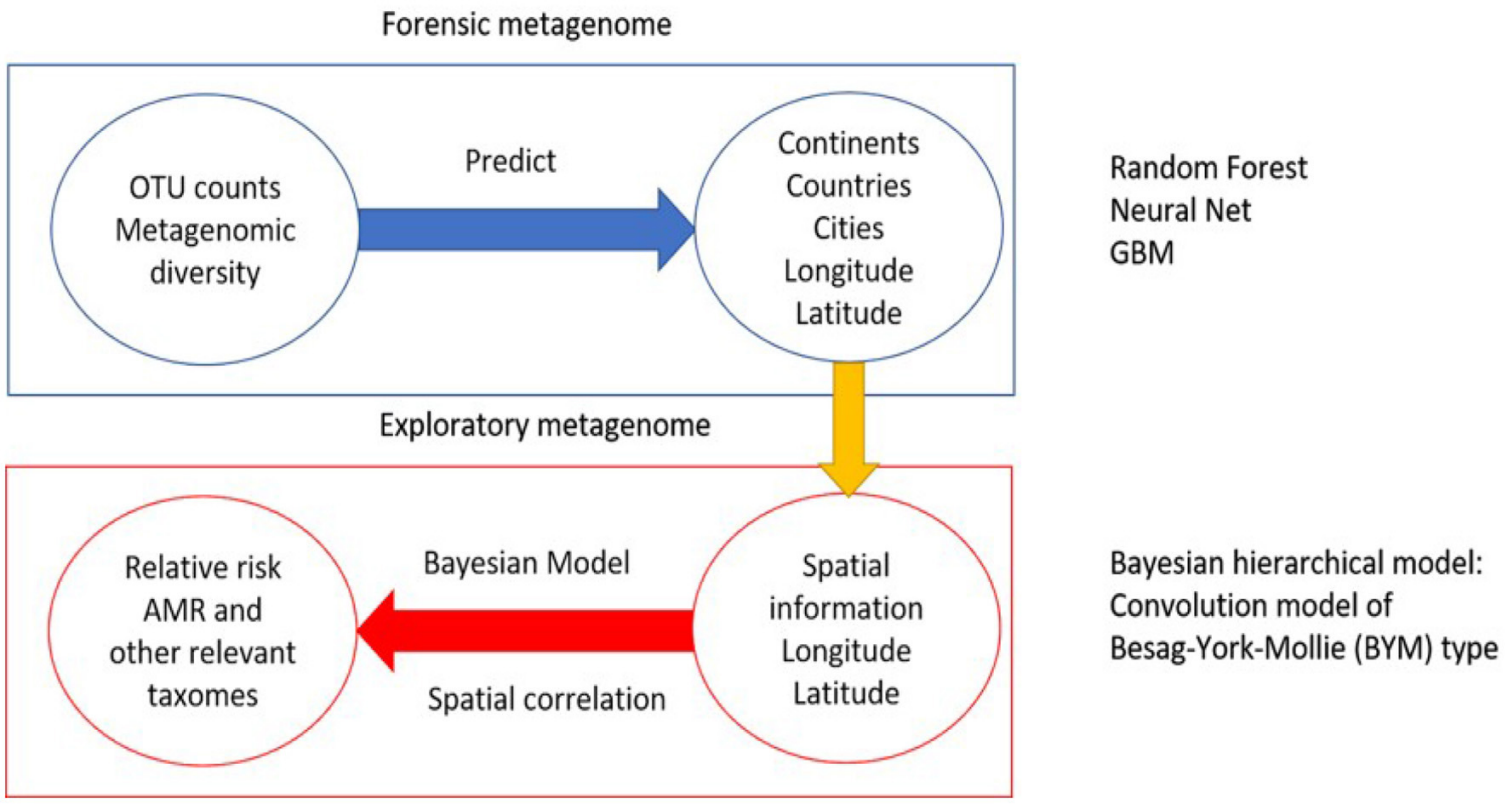
Methodology of the analysis. The first part is the application and comparison of machine learning techniques to predict samples origin. The second part is using the available spatial information to estimate relative risk of antimicrobial relevant taxa.

One of the main objectives of the study is to use the multi-source data set provided from MetaSUB/CAMDA to predict origin locations of new samples. A number of machine learning methods are used for prediction of unknown geolocations. Most of these are supervised techniques. In previous CAMDA studies, those classification methods achieved good accuracy when the mystery samples were from origins used in the training sets. In this work we compare three such approaches: Gradient Boosting Machine, Random Forest, and Neural Network. In addition, our study adds a new component to the standard analysis by using the spatial information to estimate relative risk of antimicrobial resistance. We apply a Bayesian hierarchical model to find regions with elevated relative risk of antimicrobial resistant taxa. A schematic representation of the methods are shown in Figure 1.

## 2. Materials and Methods

### 2.1. Data, Preprocessing, and Derivation of Operational Taxonomic Unit Counts

MetaSUB Consortium has more than 4,000 samples across 60 cities. Sampling took place at four major time points: a pilot study in 2015–2016 and global city sampling days (gCSD) in 2017 and 2018 with most samples taken on June 21st. Each sample was sequenced with an average of 6M 125 bp paired-end reads using Illumina NGS sequencers. CAMDA2020 challenge consists of more than 1,000 files with pair reads from 23 cities across different continents for sampling days CSD16 and CSD17. The data across cities and continents are summarized in Supplementary Table 1. Meta data for each sample and for each city include spatial information, weather data (temperature, pressure, precipitation, humidity) and demographics (population, population density, type of settlement). The data also include 121 mystery samples which origins have to be predicted.

We use Trimmomatic v0.39 (Bolger et al., 2014) to trim and filter the raw reads so that the minimum length is 50 and the average quality in a window size of 3 is no <20. We constructed a fasta file with protein sequences based on antimicrobial resistance data from NCBI (NDARO, 2020). The sequences represent genes in annotated bacteria or plasmid taxa which are associated with resistance to antibiotics or other antimicrobial drugs. Each sequence is also associated with a NCBI TaxonID. This fasta file is then used as a reference database in the classification of the reads with the Kaiju metagenomic classifier (Menzel et al., 2016). For the final summary we report the operational taxonomic unit (OTU) counts on the levels of “species,” i.e., the count is the summation of abundances of the genes corresponding to that taxon. The total number of found species related to AMR was 445. In addition to AMR taxa we use a larger database called proGenomes (Mende et al., 2017) which consists of 87,920 annotated bacterial and archaeal genomes from over 12,000 species. The total number of species found in the samples using this database is 4,973. We transformed the raw counts data to “reads per kilobase per million mapped reads” (RPKM) by normalizing them to the total number of reads for each sample. Due to the sampling date batch effect we analyze the two sampling days CSD16 and CSD17 separately.

### 2.2. Origin Prediction of Samples

Large scale metagenomics studies (Turnbaugh et al., 2007; Suzuki and Worobey, 2014; Mason et al., 2016; Casimiro-Soriguer et al., 2019) are part of a global initiative to study and understand microbiome diversity. High-throughput screening such as shotgun whole genome sequences identifies genetic information to more detailed levels such as the level of species and can further detect abundance of eukaryotes, fungi, and viruses. Most methods for analysis of metagenomics sequence data are based on the supervised machine learning techniques (Paulson et al., 2013; Wood and Salzberg, 2014; Lu et al., 2017; Delgado-Baquerizo et al., 2018). Random forest models are often used in predicting geographical locations of the samples (Fisman et al., 2014; Delgado-Baquerizo et al., 2018). Most of those models are limited to predicting samples from locations that are part of the training sets. For predicting new origins (Chen and Tyler, 2020) used Lasso regularization (Friedman et al., 2010) and Simpson's diversity index (Simpson, 1949) and incorporated previous results of association between human genetics and geographical locations. Recently more complex models have been developed for classification of protein sequence data such as deep learning (Do et al., 2020), recurrent and convolution neural networks (Le, 2019; Le and Huynh, 2019). Authors used different measures such as sensitivity, specificity, accuracy, AUC, Matthews correlation coefficient to compare the performance of the methods.

The classification of samples by their origin is commonly performed by supervised machine learning methods which involve dividing the samples into training and testing sets. In this work we did preliminary review of some of the well-known methods and decide to focus on three of them that do not involve many parameters and are easy to run within the framework of R. In particular we use Gradient Boosting Machine (GBM) (Kuhn et al., 2020), Random forest (Friedman, 2001), and Neural network (NNet) (Tin Kam Ho, 1998) as implemented in the R 3.6.3 package *caret* (Cuntz et al., 2010). We applied the above machine learning models to predict which continent and which city the samples belong to. We split our training data into two subsets: 60 and 40% and then compare the prediction results on the test set. To avoid the batch effect we analyze the samples separately by the sampling day. Recursive feature elimination (RFE), a commonly used feature selection method that fits a model and removes the weakest features, is used to screen for the top features which are then used in the prediction of continents and cities. Since the antimicrobial database has a limited number of taxa, proGenomes is preferred for the prediction part of the analysis.

### 2.3. Estimation of Relative Risk Using Spatial Modeling

Spatial autocorrelation is very common when observations that are close in space have similar values. A proportion of this spatial autocorrelation may be modeled by known covariate risk factors in a regression model, but it is common for spatial structure to remain in the residuals after accounting for these covariate effects. Spatial models such as Bayesian hierarchical models are then used to expand the linear predictor with a set of spatially autocorrelated random effects depending on the neighborhood structure of geographic areas. The random effects are typically represented with a conditional autoregressive (CAR) (Lawson, 2018) prior which induces spatial autocorrelation through the adjacency structure of the areal units. Such models are usually used in epidemiology, e.g., diseases mapping studies (Green and Richardson, 2002; Lu et al., 2007; Ma and Carlin, 2007; Lee, 2011), but are relatively new to the area of metagenomics.

The samples in MetaSUB database are coming from different cities and different areas inside each city. For the majority of them we have spatial coordinates such as longitude and latitude. For those with missing information we can use the prediction methods to determine their locations. Previous studies (Danko et al., 2019; Ryan, 2019) have found that there are spatial correlations in the metagenome profiles for closely related samples or cities. To model the spatial correlation structure explicitly we used Bayesian hierarchical models. One of them is Besag-York-Mollie (BYM) (Besag et al., 1991), which is a convolution model with CAR prior. More specifically, this is a hierarchical Bayesian model with Poisson likelihood that contains both spatially autocorrelated and independent random effects. The model response is the Standardized Incidence Ratio (SIR = Observed O/Expected E) which can be considered as a crude estimate of the relative risk (RR). The posterior estimates of the model are estimates of the relative risk and in our settings they will be interpreted as follows: if the relative risk is higher than 1 we have an elevation of AMR in the samples compared to the expected AMR which may have health consequences. If the relative risk is smaller than 1, the AMR presence is either rare or may not pose health concerns. In more details the model is described below.

We fit the model $\theta_{i}=exp\left(X^{T}\beta +\nu_{i}+u_{i}\right)$ where θ_*i*_ is the Standardized Incidence Ratio for each sample. The expected value (*E*) can be determined by different criteria. In epidemiological settings it is proportional to the city population. In this case we can use population size, population density, median of AMR in the cities or other factors which are relevant to the abundance of AMR. Here *X* is a matrix of covariates, β is the vector of regression coefficients, ν_*i*_ are spatially unstructured random effects that assume normal distribution and *u*_*i*_ are the random effects that capture the spatial autocorrelation between the samples or cities using the neighboring matrix *W*. This matrix is based on geographical contiguity between the samples. In strict mathematical terms the model as described in Besag et al. (1991) is shown below: Here $u_{i}|u_{j},j\neq i,W,\sigma_{i}^{2}\sim N\left(\frac{1}{n_{i}}\underset{i\sim j}{\sum }u_{j},\frac{\sigma_{u}^{2}}{n_{i}}\right)$. In more strict mathematical terms the model is described in Lawson (2018).

$$\begin{array}{l} O_{i}|E_{i},\theta_{i}\sim Poisson\left(E_{i}\theta_{i}\right),i=1,\ldots ,n\\ \;ln\left(\theta_{i}\right)=x_{i}^{T}\beta +\nu_{i}+u_{i}\\ \;\nu_{i}|\sigma \sim N\left(0,\sigma_{\nu }^{2}\right)\\ \;u_{i}|u_{j},j\neq i,W,\sigma_{i}^{2}\sim N\left(\frac{1}{n_{i}}\underset{i\sim j}{\sum }u_{j},\frac{\sigma_{u}^{2}}{n_{i}}\right)\\ \;\beta_{j}\sim N\left(0,1000\right),j=0,\ldots ,p. \end{array}$$

In our case *W* = 1 − *D* (Normalized Distance) between samples latitude and longitude positions. We can use both the continuous distance or convert W to a binary matrix based on a threshold, e.g., samples i and j are neighbors if the distance between them is less than a specified threshold (e.g., 1, 10 km). The response is assumed to follow Poisson distribution and it accounts for overdispersion *Var*(*O*) > *E*(*O*) and this is an advantage over the pure Poisson model.

We use the Bayesian setting implementation in R 3.6.3 package CARBayes (Lee, 2013), where inference is based on Markov chain Monte Carlo simulation. The model is fit with the function S.CARbym from the above package. Moran's *I*-test (Gittleman and Kot, 1990) was used to measure the spatial autocorrelation based on both feature locations and feature values simultaneously by evaluating whether the pattern expressed is clustered, dispersed, or random. To check which model has a better fit we looked at the Deviance Information Criteria (DIC) (Ma and Carlin, 2007). The model convergence is also checked by Geweke z-scores (Geweke, 1992). We run the models with several covariates including surface material of the samples and climate conditions such as Köppen climate classification (McMurdie and Holmes, 2014). We also generated Google Maps 2020 where we overlay the estimated relative risk so that we can identify regions with elevated AMR.

## 3. Results

### 3.1. Antimicrobial Resistance Taxa Profiles

**Figure 2:**
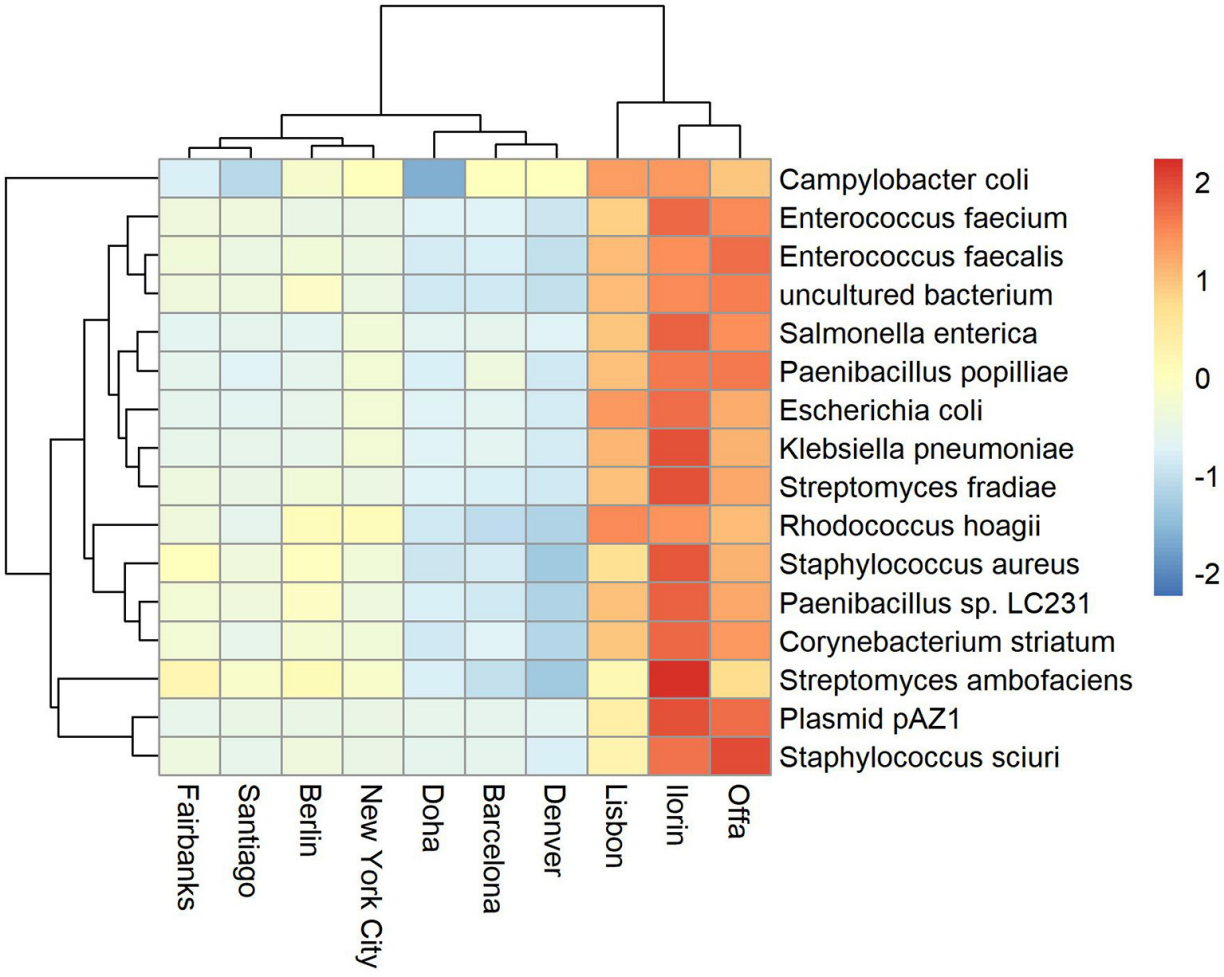
Top abundant antimicrobial resistant taxa for sampling day CSD16. The cities Offa, Ilorin, and Lisbon show high prevalence of antimicrobial resistant related taxa.

**Figure 3:**
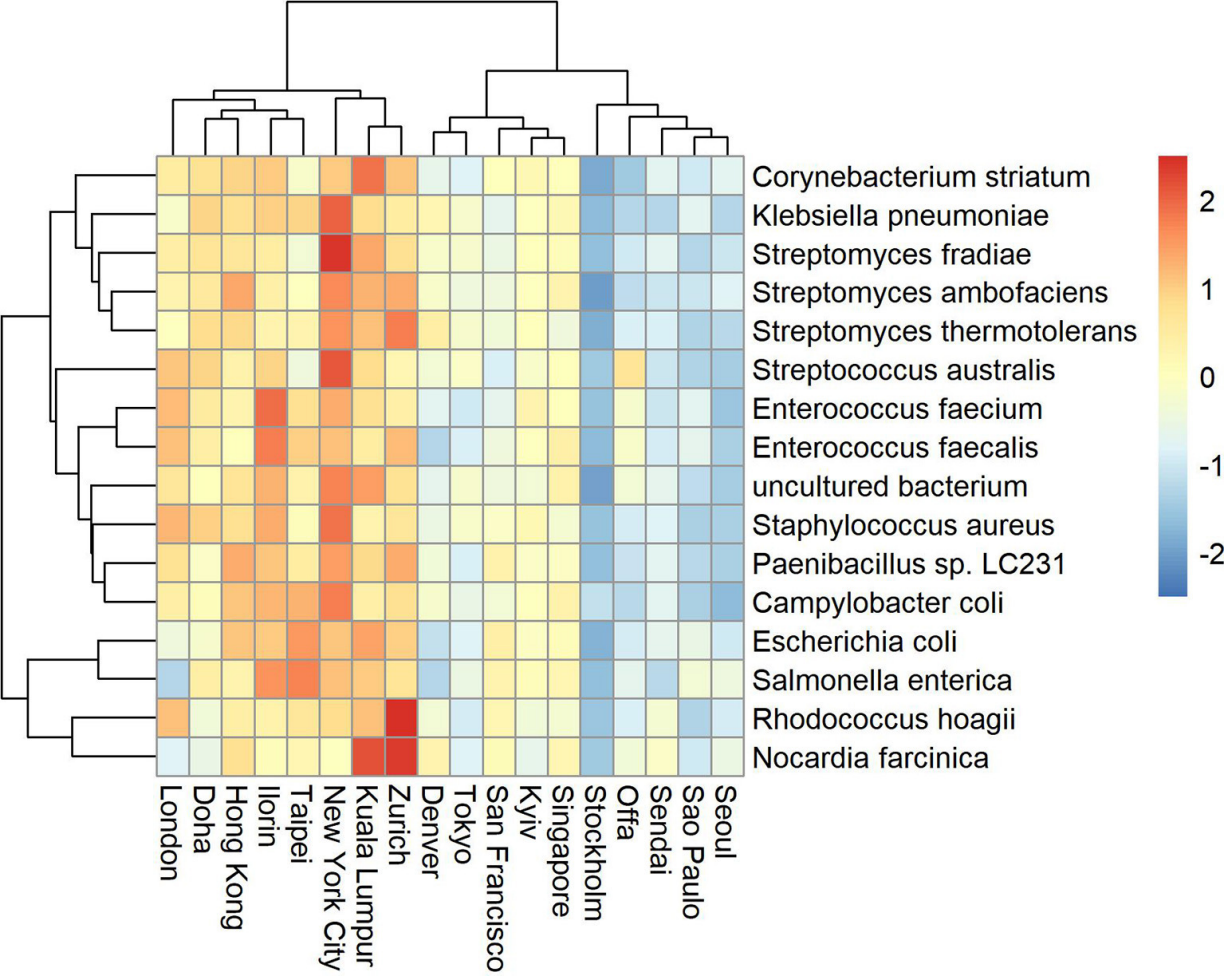
Top abundant antimicrobial resistant taxa for sampling day CSD17. Big cities such as New York, London, Hong Kong, and Kuala Lumpur show high prevalence of antimicrobial resistant related taxa.

Antimicrobial resistance known genes and the corresponding bacteria taxa represent a relatively small portion of the available global metagenome profile (Danko et al., 2019). Based on the Kaiju metagenomic classifier, who uses modified backward search on a memory-efficient implementation of the Burrows-Wheeler transform, we also found that the relative abundance of antimicrobial related species represent on average between 0 and 0.33 of the total reads. Some cities showed higher variability and counts such as Fairbanks (max 0.28), Lisbon (max 0.2), Ilorin (max 0.33). The top abundant antimicrobial related taxa are shown in Figures 2, 3. One of the clusters includes *Salmonella enterica, Staphylococcus aureus*, and *Escherichia coli* which show high abundance in Ofa, Ilorin, and Lisbon. The antimicrobial genes in *Streptomyces* related classes are more prevalent in the big cities such as London, New York, Hong Kong, and Kuala Lumpur. Samples from Berlin, Tokyo, Stockholm, and Doha have small or zero counts among the top abundant antimicrobial taxa.

Some of the strongest correlations of the median antimicrobial related taxa (correlation > 0.6, *p* < 0.01) with weather data across cities are: different measures of humidity variability and *Vibrio parahaemolyticus*; humidity averages and *Campylobacter jejuni, Corynebacterium striatum, Paenibacillus* sp. LC231, *Rhodococcus hoagii, Streptococcus australis*, and *Streptomyces ambofaciens*; temperature and *Pseudomonas aeruginosa*. *Vibrio parahaemolyticus* and *P. aeruginosa* show the strongest negative correlations to humidity and pressure variability, respectively.

### 3.2. Prediction of Sample Origins

To predict sample origin we use three common machine learning techniques: Gradient Boosting Machine (GBM), Random Forest (RF), and Neural Network (NNet). To select the best features for the models we apply Recursive feature elimination (RFE) as implemented in the R packages *caret*. This outer resampling method is based on cross-validation resampling with 10-fold and was repeated 3 times. The k-fold approach involves dividing the set into k groups or folds of approximately equal size. The first set is treated as a validation set and the method is fit on the remaining k-1 groups, where k is usually taken to be equal to 5 or 10. Table 1 shows the accuracy if different numbers of features are selected. Adding more that 16 features does not improve significantly the accuracy so for our future analysis we focused on the top 16 features. The top features for antimicrobial taxa for CSD16 and CSD17 sampling days are: *S. enterica, E. coli, Paenibacillus* sp. LC231, *S. aureus, Campylobacter coli, Streptomyces fradiae, Klebsiella pneumoniae, Enterococcus faecium*. The top features when using proGenomes taxa are: *Vibrio cyclitrophicus, Sphingomonas elodea, Bacillus azotoformans, Acinetobacter indicus, Methylobacterium radiotolerans, Lactobacillus fermentum*.

**Table 1:** Accuracy of recursive feature elimination (RFE) for Antimicrobial resistant taxa counts for CSD16 and CSD17 sampling days and for proGenomes for CSD17.

	**AMR database**				**proGenomes database**	
	**CSD16**	**CSD16**	**CSD17**	**CSD17**	**CSD17**	**CSD17**
	**Continent**	**City**	**Continent**	**City**	**Continent**	**City**
Features (4)	0.55	0.43	0.45	0.26	0.63	0.35
Features (8)	0.59	0.49	0.50	0.34	0.73	0.58
Features (16)	0.63	0.54	0.55	0.40	0.78	0.69
Features (all)	0.62	0.55	0.58	0.48	0.77	0.75

*The number of total features are: AMR CSD16—394, AMR CSD17—415, and proGenomes—4973*.

Next we compare the three machine learning methods GBM, Random Forest and Neural Network using the counts from proGenomes database. The methods are available in the R package *caret* with the option method = “gbm,” method = “rf,” and method = “nnet.” The Gradient boosting option runs the so called stochastic gradient boosting. The final parameters are: N Boosting Iterations = 150, Max Tree Depth = 6, Shrinkage = 0.1, and Minimum Terminal Node Size = 10, where the Shrinkage and Minimum Terminal Node Size were kept at 0.1 and 10, respectively while tuning. The Neural network version is feed-forward neural network with a single hidden layer as implemented in the R package “nnet” with size = 19 and decay = 0.04216965. The number of Randomly Selected Predictors in Random Forest was 3. The source code for our analysis as provided in the Supplementary Material allows to run additional models including more complex neural networks provided in the R package *caret*. The possible models can be selected from here http://topepo.github.io/caret/available-models.html. We divided the data into training (60%) and testing (40%) sets and use RFE to select the best features. First we compare the prediction results (accuracy and 95% confidence intervals) using antimicrobial taxa [NCBI annotated (NDARO)] and proGenomes (Mende et al., 2017) count data (see Table 2). As expected we obtain much better prediction accuracy using the larger proGenomes database. Table 3 lists the prediction results (accuracy and 95% confidence intervals) of the three methods using the top 16 features. The methods show similar performance with both Random forest and Stochastic Gradient Boosting outperforming Neural Network method. Random Forest shows better predictability for cities while Stochastic Gradient Boosting shows better performance for continents when using AMR, but with proGenomes both methods achieve the same results.

**Table 2:** Accuracy and 95% confidence intervals for Random Forest prediction with the top 16 features selected using Recursive feature elimination (RFE).

	**CSD17**	
	**Continent**	**City**
AMR	0.55 (0.48,0.61)	0.46 (0.4,0.52)
proGenomes	0.81 (0.76,0.86)	0.71 (0.65,0.76)

*The top predicted cities using AMR count taxa are Stockholm, Taipei, and New York City (balanced accuracy 88, 88, and 85%, respectively) while the top predicted with proGenomes count data are Kuala Lumpur, London, and New York City with almost perfect accuracy*.

**Table 3:** Accuracy and 95% confidence intervals for Gradient Boosting Machine (GBM), Random Forest and Neural Network (Nnet) predictions with the top 16 features selected using Recursive feature elimination (RFE).

	**CSD17**	
	**Continent**	**City**
GBM(AMR)	0.61 (0.55,0.67)	0.40 (0.34,0.47)
Random Forest (AMR)	0.55 (0.48,0.61)	0.46 (0.4,0.52)
Neural Net (AMR)	0.51 (0.45,0.57)	0.38 (0.32,0.44)
GBM (proGenomes)	0.82 (0.77,0.87)	0.67 (0.61,0.72)
Random Forest (proGenomes)	0.81 (0.76,0.86)	0.71 (0.66,0.76)
Neural Net (proGenomes)	0.78 (0.73,0.83)	0.60 (0.54,0.66)

*For each method the tuning parameters are selected such that the best accuracy is achieved*.

Samples from London, New York, Kuala Lumpur display high prediction balanced accuracy, both sensitivity and specificity (>95%), while samples from others like Doha and Kiev have poor sensitivity (<40%) even when using a larger database such as proGenomes. One of the reasons may be the smaller sample size for cities as Doha for CSD17. For example using only AMR taxa for CSD16 we can achieve accuracy of 83% for Doha mostly because we have 50 samples for this day. However, the sample size can not completely explain the prediction results since cities with one of largest numbers of samples such as Hong Kong and Kiev still have low accuracy. Samples from Kiev for example are misclassified as samples from Zurich, while samples from Hong Kong are often misclassified as ones from Singapore, Taipei, or Tokyo. Close cities like Ilorin and Offa show similar profiles and are difficult to differentiate. When combined and considered as one city the sensitivity and accuracy for them increases and becomes greater than 90 percent. Therefore, the best prediction is achieved for Sub Saharan Africa (combined Ilorin and Offa samples are correctly classified) followed by North America while Middle East and South America have the lowest classification accuracy. Samples from Middle East are often misclassified as samples from East Asia, while half of the samples from South America were misclassified as samples from Europe and East Asia. The prediction results by city and by continents, sorted by balanced accuracy are listed in Tables 4, 5.

**Table 4:** Cities prediction statistics for Random Forest for CSD17 sampling date using proGenomes database with the top 16 features from RFE.

	**CSD17**		
	**Sensitivity**	**Specificity**	**Accuracy**
Kuala Lumpur	1.00	1.00	1.00
London	1.00	0.992	0.996
New York	0.90	1.00	0.95
Sendai	0.92	0.98	0.95
Stockholm	0.90	0.96	0.93
Seoul	0.84	0.98	0.91
San Francisco	0.89	0.99	0.90
Ilorin	0.79	0.99	0.89
Taipei	0.80	0.97	0.88
Singapore	0.68	0.96	0.82
Tokyo	0.60	0.98	0.79
Denver	0.50	0.99	0.74
Hong Kong	0.53	0.95	0.74
Zurich	0.46	0.99	0.72
Kiev	0.42	0.98	0.70
Offa	0.40	0.996	0.70
São Paulo	0.36	0.99	0.68
Doha	0.33	1.00	0.67

*The overall accuracy is 0.71 (0.65, 0.76). The data are sorted by balanced accuracy which is the average between sensitivity and specificity. For each method the tuning parameters are selected such that the best accuracy is achieved*.

**Table 5:** Continents prediction statistics for Random Forest for CSD17 sampling date using proGenomes database with the top 16 features from RFE.

	**CSD17**		
	**Sensitivity**	**Specificity**	**Accuracy**
Sub Saharan Africa	1.00	1.00	1.00
North America	0.90	0.99	0.95
East Asia	0.89	0.79	0.84
Europe	0.69	0.91	0.80
South America	0.45	0.996	0.73
Middle East	0.17	1.00	0.58

*The overall accuracy is 0.81 (0.76, 0.86). The data are sorted by balanced accuracy which is the average between sensitivity and specificity*.

**Figure 4:**
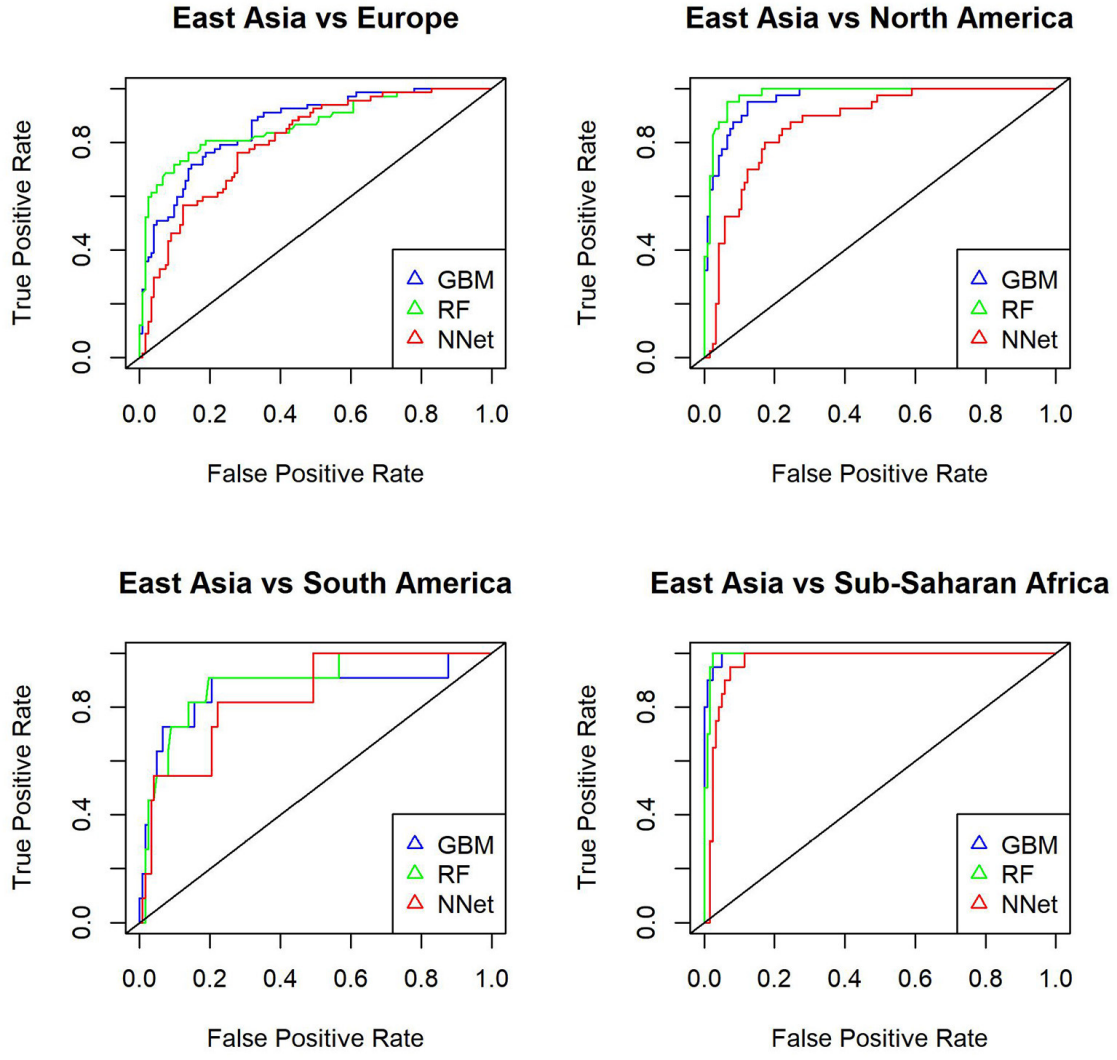
Multiple ROC curves calculated with R package pROC for comparison of continent predictions with GBM (gbm), Random Forest (rf) and Neural Network (nnet) from R package *caret*. The multi-class areas under the curve are 0.96, 0.956, and 0.926, respectively.

Next we use the three methods to predict the mystery samples. The current methods can not predict origin of samples that do not belong to the training set so when predicting cities we need to exclude mystery cities that were not present in the training set. Accuracy for Hong Kong, Kiev, Taipei, Tokyo, and Zurich are as follows: GBM (0.64,0.66,0.78,0.66,0.69), RF (0.69,0.69,0.84,0.78,0.77), NNet (0.65,0.63,0.66,0.63,0.63), respectively. Accuracy for East Asia, Europe, and South America are as follows: GBM (0.81,0.69,0.49), RF(0.8,0.65,0.49), NNet (0.73,0.67,0.53). Random Forest has the best performance when predicting cities while all three methods have similar performance when predicting continents. In more details Tables 6, 7 show the accuracy for mystery samples with Random Forest. Samples from East Asia show about 80% accuracy while samples from Europe have 65% accuracy with the rest of the samples misclassified as samples from North America or East Asia. Samples from South America are also misclassified as samples from Europe or North America. Focusing on the cities we see that samples from Taipei and Tokyo achieved the best overall accuracy. On the other hand about one third of samples from Zurich are predicted to be from Kiev. Similarly to the CSD17 data set, Hong Kong and Kiev have the worst accuracy. Half of the Hong Kong samples are predicted as belonging to other Asian cities such as Taipei and Tokyo. The samples from Kiev are also misclassified as either samples from Singapore or Zurich. Again we observe that cities in the same continent share similar profiles and can not be always differentiated. This is especially true for East Asia where the accuracy is above 80% and the samples are rarely misclassified as belonging to another continent. Comparison of the multi ROC curves calculated with R package pROC for prediction of continents is shown in Figure 4. As described above the GBM and RF have higher accuracy and in this case also higher multi-class areas under the curve compared to the simple neural network method.

**Table 6:** Confusion matrix for prediction of cities membership of the mystery samples using Random Forest with proGenomes taxa data.

	**City**				
	**Hong Kong**	**Kiev**	**Taipei**	**Tokyo**	**Zurich**
Denver	1	0	0	0	0
Hong Kong	6	0	1	0	0
Ilorin	1	0	0	0	0
Kiev	1	5	0	1	2
New York	0	0	2	0	0
San Francisco	0	0	0	0	1
São Paulo	1	0	0	1	0
Sendai	0	0	0	1	0
Seoul	1	1	0	1	1
Singapore	0	3	0	0	2
Stockholm	1	0	0	1	0
Taipei	2	0	8	1	0
Tokyo	1	0	0	8	0
Zurich	0	2	0	0	8
Accuracy	0.69	0.69	0.84	0.78	0.77

**Table 7:** Confusion matrix for prediction of continents membership of the mystery samples using Random Forest with proGenomes taxa data.

	**Continent**		
	**East Asia**	**Europe**	**South America**
East Asia	33	18	0
Europe	5	40	10
North America	0	11	2
South America	2	0	0
Accuracy	0.80	0.65	0.49

**Figure 5:**
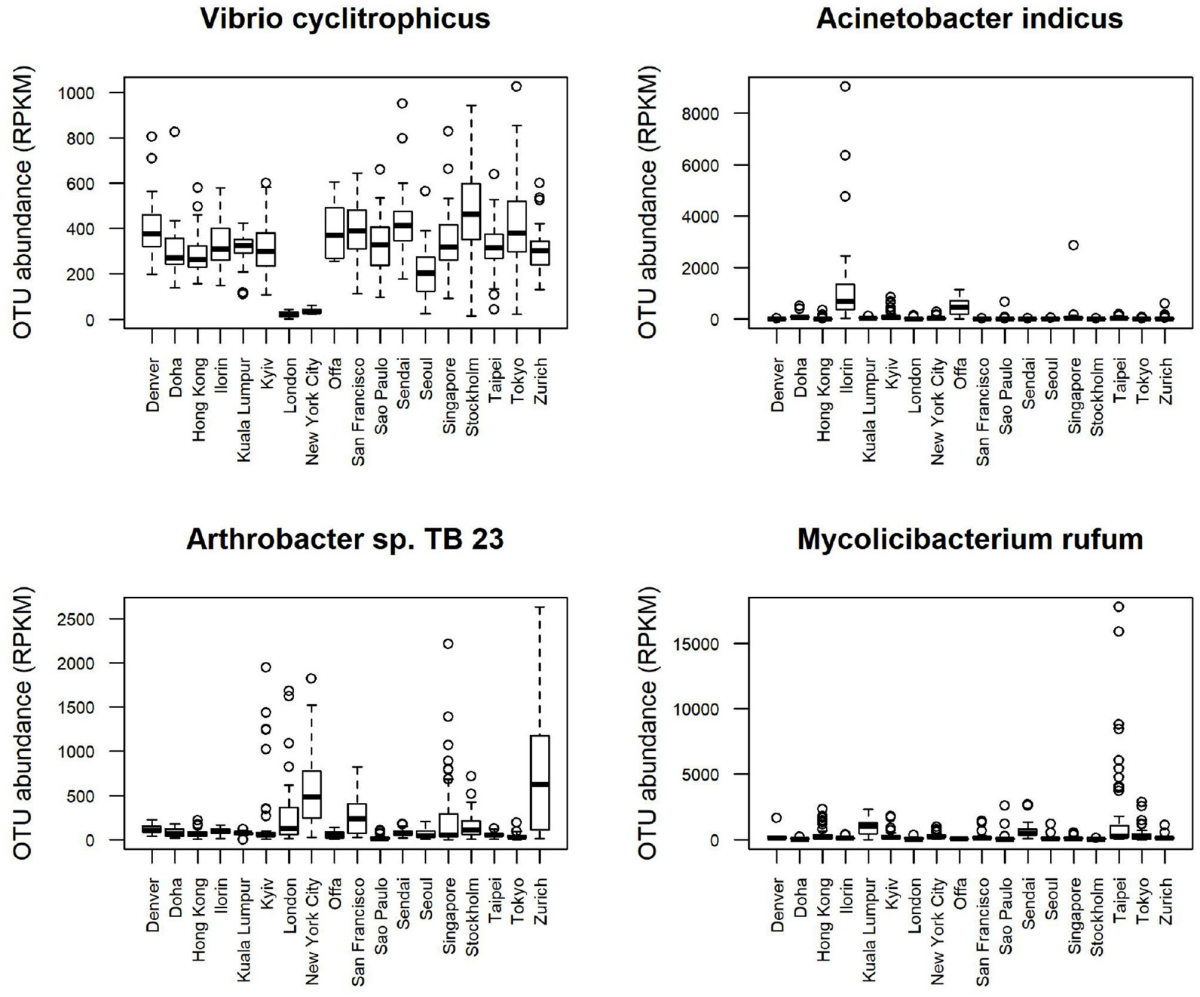
Boxplot of the top features selected using Recursive feature elimination (RFE) with proGenomes reference database for CSD17 sampling day.

**Figure 6:**
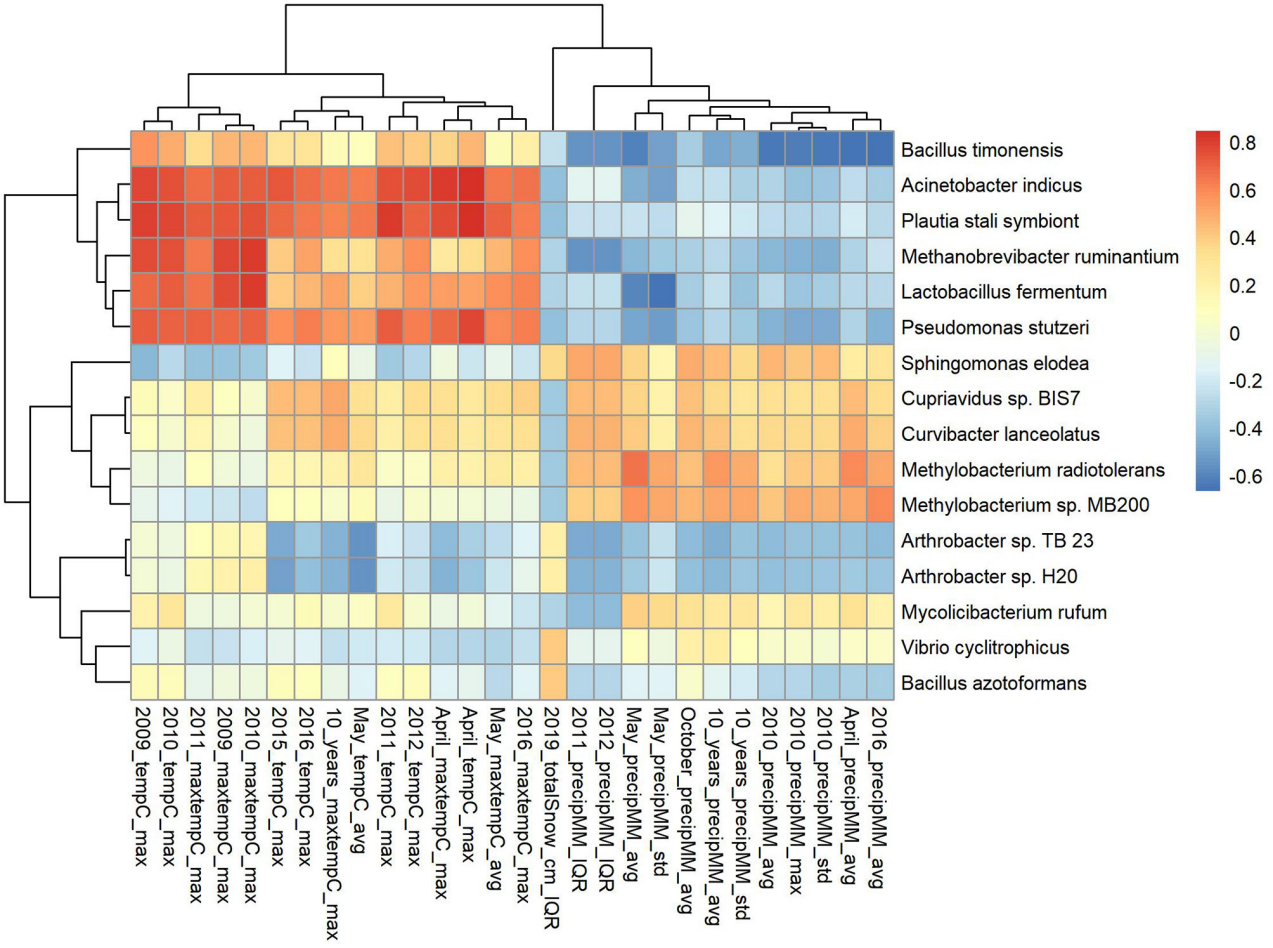
Correlation of the top 16 features (selected using Recursive feature elimination (RFE) with proGenomes reference database for CSD17 sampling day) with climate conditions.

Boxplots of average abundance across cities for some of the top features are shown in Figure 5. London and New York have similar profiles for many of them, e.g., low counts *V. cyclitrophicus* but high counts for *Arthrobacter* sp. Tb23. Ilorin and Offa show high relative abundance for opportunistic pathogens such as *A. indicus* and *Bacillus boroniphilus*. The correlations of the top 16 features with the climate conditions are shown in Figure 6. There are two major clusters with opposite correlations. The first cluster has positive correlations with measures of temperature and negative correlations with pressures, while the second cluster shows the inverse pattern.

### 3.3. Spatial Modeling

For our spatial analysis we use all available abundant genes to apply the convolution model. We checked the spatial correlation in the cities using Moran's *I*-test. Cities such as New York (max 0.44, *p* < 0.01), Ilorin (0.38, *p* < 0.01), Hong Kong (0.41, *p* < 0.01), and Taipei (0.6, *p* < 0.01) show strong spatial correlations for many of the above antimicrobial resistant taxa. Metagenomics count data often show overdispersion (McMurdie and Holmes, 2014) since they are heterogeneous due to the different cities and countries. We performed a formal overdispersion test for the top 16 antimicrobial features from the prediction models by fitting a Poisson model with covariates and using ordinary least square regression to estimate the parameter for overdispersion (Kleiber and Zeileis, 2008). The results show that all except one of the top antimicrobial features exhibit overdispersion with *p*-values well below 0.01.

**Figure 7:**
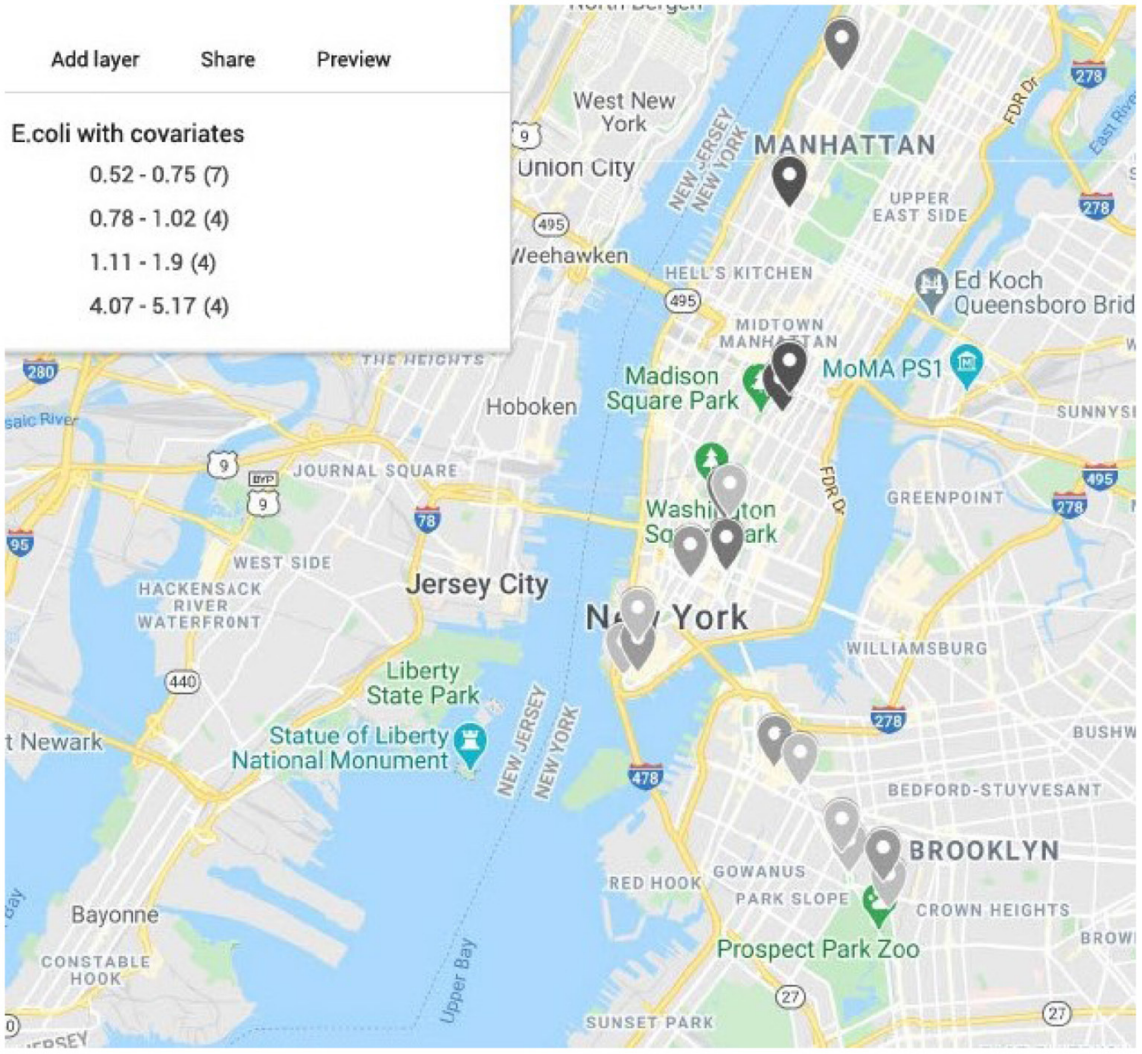
Map of New York City generated from latitude and longitude of the samples from CSD16 sampling day using Google Maps. Color represents the estimated relative risk from the model for *E. coli* with area population and density as covariates. Darker color shows higher relative risk areas compared to the median across all samples in the city for CSD16. Most of the high risk areas are in Manhattan where density of the population is higher. Area density has a significant effect in the model.

**Figure 8:**
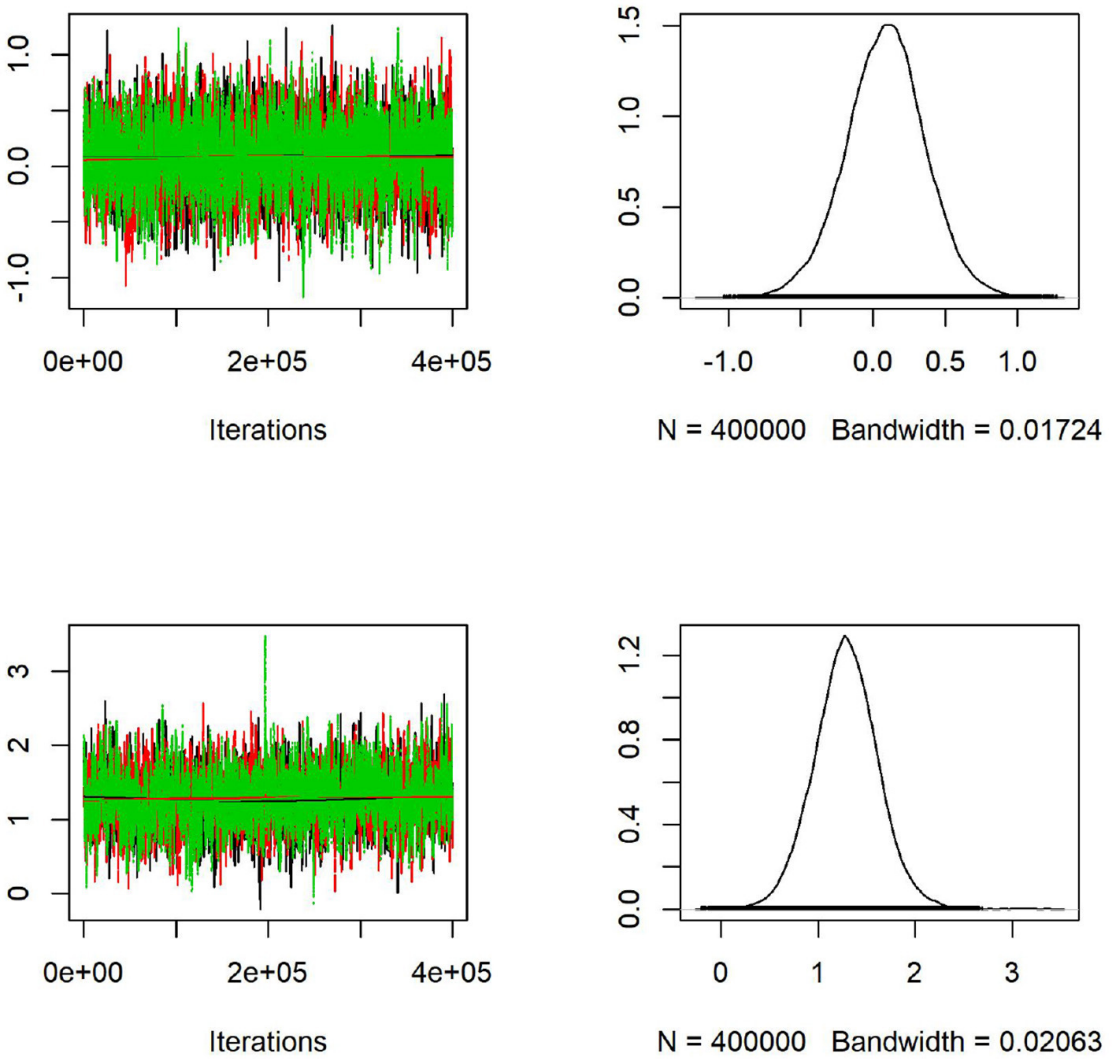
Convergence of the Markov chains: trace **(left)** and posterior density **(right)** plots for the regression parameters of population (first row) and density (second row) as covariates in the spatial model applied to CSD16 samples from New York City. Density has a significant effect on the *E. coli* resistant genes abundance as shown in the graph with 95% confidence interval not containing zero.

We generate maps using Google Map 2020 based on the latitude and longitude information from MetaSub and overlaid the results from the convolution Baeysian spatial model. They include the SIR ratio (Standardized Incidence Ratio = Observed AMR counts/Expected AMR counts) and the estimated posterior estimates of the Relative risk (RR). Here the expected value is the median across the considered area. Darker colors represent areas with higher AMR relative risk compared to the median risk in the city. Population density as a covariate in the model has a significant effect for the relative risk in New York City as shown in Figure 7 with most dense areas in Manhattan having the largest abundance of resistant *E. coli* taxa. In Figure 8, we plot the model convergence of the Markov chains: trace and posterior density plots for the regression parameters of the covariates (population and density) in New York areas.

**Figure 9:**
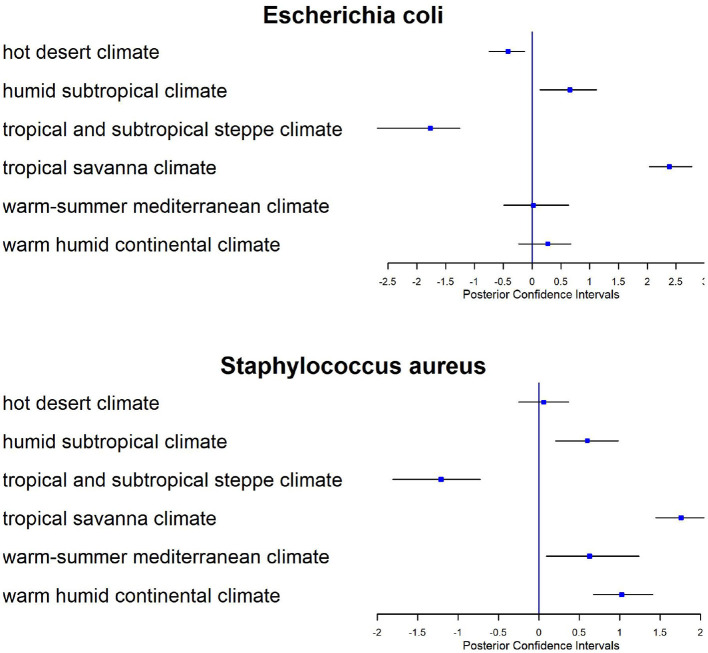
Forest plots for posterior estimates of Köppen climate classification as a covariate for CSD16 sampling day for two antimicrobial related taxa: *E. coli* and *Staphylococcus aureus*.

Climate conditions play an important role in metagenomics profiles and we tested their effects on the relative risk. In Figure 9, we show the posterior estimates from the model of the regression parameters associated with Köppen climate classification for *E. coli* and *Staphilococus aureus*. Here we applied the model to all CSD16 sampling day data using the distance matrix based on latitude and longitude of the samples. The tropical and subtropical steppe climate has negative effects (confidence intervals are less than zero), i.e., reduces the abundance of *E. coli* while tropical savanna climate is positively associated (confidence intervals are greater than zero) with both bacteria. On the other hand we observe no effect of Köppen climate classification or other factors on antimicrobial resistant genes related to *Streptococcus pneumonia, Salmonella* sp., *Mycobacterium tuberculosis*, or *Enterobacter* sp. since the sequences related to such resistance have very limited abundance in the data.

## 4. Discussion

In this work we show that the three machine learning methods namely Gradient Boosting machine, Random Forest and Neural Network have similar predictive power to classify the origin of the samples. Using a large database such as proGenomes that contain more than 80,000 annotated bacterial and archaeal genomes we achieve high accuracy (up to 80%). Due to the larger number of samples for CSD17 sampling day we performed the comparison between predictions using different databases and different machine learning techniques on those samples. Some cities are well predicted such as London, New York, and Kuala Lumpur while samples from others like Doha, Singapore, and Kiev are poorly classified. Continents such as Sub Saharan Africa, North America, and East Asia have the highest sensitivity as most of the samples were correctly predicted. South America and the Middle East (Doha) shows the lowest sensitivity. We observe that close cities (Ilorin, Offa) or cities in the same continent (especially East Asia) show similar profiles and often can be mistaken for each other.

We obtain much better accuracy (e.g., 81 vs. 55% for continents and 71 vs. 46% for cities) with the larger proGenomes database compared to using only NCBI AMR taxa database. Cities such as Taipei and Tokyo have the best accuracy while samples from Kiev are poorly classified and often misclassified as samples from Zurich and vice versa. Hong Kong mystery samples are also misclassified but mostly for samples from other cities in East Asia. The prediction for both East Asia (80%) and Europe (60%) is similar to the one achieved by using samples from CSD17 as a testing set (89 and 69%, respectively). Similarly South America has shown worse prediction accuracy but in the mystery samples we have Bogota while the training set has São Paulo. To improve further the prediction accuracy we can utilize additional climate metadata as covariates, use a larger than proGenomes database, different parameters in the current models and better normalization methods to combine CSD16 and CSD17 by avoiding the potential batch effect. This work focuses on easily applicable methods as a first step in order to check how good the predictions are with few parameters. The prediction accuracy of continents is high while prediction on more granular level like cities could be improved. The R package *caret* have a large number of more complex models that potentially could be further explored. Most of them involve a large set of parameters that need to be tuned and will be more helpful when trying to improve the accuracy on a finer scale and distinguish between close cities or countries. In addition, deep learning neural networks (Le, 2019; Le and Huynh, 2019; Do et al., 2020) which are recently developed to predict protein functions based on sequence data can also be useful to determine geographical origins of metagenomics data.

In addition to the prediction of origins we apply spatial models to access the risk of antimicrobial resistance inside cities and across countries. Standard regression models that do not take into account the spatial dependencies do not work well here since the parameter estimates and results will be unreliable. Moreover, the data show overdispersion and ordinary linear regression models will produce biased estimates. Therefore, applying spatial models in particular Bayesian hierarchical models is relevant. More spatial information and sampling of closely located cities and countries will help to build better and more detailed maps of AMR relative risk. The models can be further applied to include multiple covariates (climate conditions such as temperature, pressure). In some cases it may be appropriate to consider a negative binomial model, instead of Poisson. For AMR taxa with excessive zeros we can use instead a zero-inflated Poisson model in the same framework as above assuming that the response follows a zero-inflated Poisson distribution which is a mixture of a point mass distribution based at zero and a Poisson distribution. The current model BYM is a globally smooth CAR model, but this is not always the case. Instead we can consider locally smooth methods (Leroux et al., 2000; Lee and Mitchell, 2012; Lee and Sarran, 2015). The maps such as shown in Figure 7 can be used to assess the risk for each area for the presence of AMR related taxa which may impact public health decisions.

Understanding the risk profiles has practical implications both in short and long term. It is widely accepted that while Antimicrobial resistant genes may be present, for the establishment of a resistance population there is a need for a beneficial ratio between selection pressure and fitness cost (Hiltunen et al., 2017). Environmental factors, city design amongst them, are a major force defining this ratio (Okeke and Edelman, 2001). The models we generated can help to develop a better understanding of the process through which Antimicrobial resistance is established by providing critically important environmental parameters. Ultimately, we may be able to alter some of these parameters in order to control the Antimicrobial resistance establishment as part of the One health concept (Pieri et al., 2020). One could also speculate that longitudinal data of well defined geographic regions risk profiles, as it will inevitably become available (Bengtsson-Palme et al., 2018), can be used to evaluate the effectiveness of the public policy decisions.

We emphasize that using a relative risk (RR) as a new measure for AMR and incorporating the spatial information for the samples as defined by their longitude and latitude could lead to better prediction and understanding of risk posed by the surrounding microbial communities. In our future work we will extend the analysis by considering a broader class of models including spatio-temporal models.

The count OTU data and the source codes in R can be found in Supplementary Material for this paper. The programs generate all the tables and figures so the results can be reproduced. In addition the code allows the users to change the parameters, for example using different set of tuning, no tuning option and also to run additional machine learning methods as provided in the package *caret* and further improve the results. The Supplementary Material includes two compressed files with the data and codes respectively and a pdf file which describes the content of the data files and the R source codes.

## Data Availability Statement

The original contributions presented in the study are included in the article/Supplementary Material, further inquiries can be directed to the corresponding author/s. The Supplementary Material includes two compressed files with the data and codes respectively and a pdf file which describes the content of the data files and the R source codes.

## Author Contributions

MZ, RY, and DV wrote the paper. RY and MZ analyzed the data. ST and DV collect the data. IM integrate and preprocess the data. SK preprocessed the data and wrote parts of the text. DD and CM provided the final dataset and participated in the text writing and logistics. All authors contributed to the article and approved the submitted version.

## Conflict of Interest

SK was employed by company Bristol-Myers Squibb, NJ. The remaining authors declare that the research was conducted in the absence of any commercial or financial relationships that could be construed as a potential conflict of interest. The handling editor declared a past collaboration with the authors DD and DV.
