# Supplementary material for: Origin Sample Prediction and Spatial Modeling of Antimicrobial Resistance in Metagenomic Sequencing Data
Source: Front Genet. 2021 Mar 4;12:642991. doi: 10.3389/fgene.2021.642991 (PMC7983949; doi:10.3389/fgene.2021.642991)
Supplement: Supplementary file 2 [file Data_Sheet_2.pdf]

Supplementary Material

1 SUPPLEMENTARY DATA

The following files contain OTU (operational taxonomic units) count data of RPKM (reads per kilobase per million mapped reads) data using AMR and proGenomes reference database in kaiju classifier.

AMR_counts.csv:
Counts derived from kaiju using the AMR database from NCBI as a reference for CSD16 and CSD17 samples
proGenome_counts.csv:
Counts derived from kaiju using proGenomes database as a reference for CSD17 samples
Mystery_counts.csv:
Counts derived from kaiju using proGenomes database as a reference for Mystery samples

The following files contain metadata associated with the samples

Metadata_AMR.csv:
Metadata for AMR (Antimicrobial resistance) counts samples for CSD16 and CSD17 samples
Metadata_proGenomes.csv:
Metadata for proGenomes counts for CSD7 samples
Metadata_mystery.csv:
Metadata for proGenomes counts for mystery samples
Weather.csv:
Weather related metadata
NYC_meta.csv:
Population and density of New York City by Zip code queried from
http://www.usa.com/rank/new-york-state–population-density–zip-code-rank.htm
amr.fmi: AMR database used in kaiju. The accession numbers are from
https://www.ncbi.nlm.nih.gov/pathogens/antimicrobial-resistance/

The following files contain R scripts used in the analysis. Packages required to run the analysis are: ape, CARBayes, caret, geosphere, forestplot, pheatmap, RANN

Spatial.R:
Bayesian hierarchical Model (BYM) using CARBayes package
Moran.R:
Testing for spatial correlation using Moran's I test
Predict. R:
Prediction using GBM, Random Forest and NNet with caret package
Tables.R:
Wrapper code to generate the Tables in the paper
Figures.R:
Wrapper code to generate the Figures in the paper

Predictors.Rdata:
Features from the Recursive feature elimination (RFE) method

2 SUPPLEMENTARY TABLES AND FIGURES

2.1 Tables

	Sampling Day	
	CSD16	CSD17
Barcelona	38	
Berlin	37	
Denver	23	22
Doha	50	15
Fairbanks	42	
Ilroin	25	36
Lisabon	19	
New York	33	50
Offa	9	14
Santiago	13	
Stockholm		50
Hong Kong		49
Seoul		48
Kiev		48
Kuala Lumpur		30
London		37
Sao Paulo		29
San Francisco		29
Singapore		48
Taipei		50
Tokyo		50
Sendai		32
Zurich		33

Table S1. Number of samples analyzed for each of the sampling days CSD16 and CSD17.
